# An eight-mRNA signature outperforms the lncRNA-based signature in predicting prognosis of patients with glioblastoma

**DOI:** 10.1186/s12881-020-0992-7

**Published:** 2020-03-19

**Authors:** Zhenyu Gong, Fan Hong, Hongxiang Wang, Xu Zhang, Juxiang Chen

**Affiliations:** Department of Neurosurgery, Changzheng Hospital, Second Military Medical University, NO. 415 Fengyang Road, Huangpu Distinct, Shanghai, 200003 China

**Keywords:** Glioblastoma, mRNA, lncRNA, Prognosis, Risk score

## Abstract

**Background:**

The prognosis of the glioblastoma (GBM) is dismal. This study aims to select an optimal RNA signature for prognostic prediction of GBM patients.

**Methods:**

For the training set, the long non-coding RNA (lncRNA) and mRNA expression profiles of 151 patients were downloaded from the TCGA. Differentially expressed mRNAs (DEGs) and lncRNAs (DE-lncRNAs) were identified between good prognosis and bad prognosis patients. Optimal prognostic mRNAs and lncRNAs were selected respectively, by using univariate Cox proportional-hazards (PH) regression model and LASSO Cox-PH model. Subsequently, four prognostic scoring models were built based on expression levels or expression status of the selected prognostic lncRNAs or mRNAs, separately. Each prognostic model was applied to the training set and an independent validation set. Function analysis was used to uncover the biological roles of these prognostic DEGs between different risk groups classified by the mRNA-based signature.

**Results:**

We obtained 261 DEGs and 33 DE-lncRNAs between good prognosis and bad prognosis patients. A panel of eight mRNAs and a combination of ten lncRNAs were determined as predictive RNAs by LASSO Cox-PH model. Among the four prognostic scoring models using the eight-mRNA signature or the ten-lncRNA signature, the one based on the expression levels of the eight mRNAs showed the greatest predictive power. The DEGs between different risk groups using the eight prognostic mRNAs were functionally involved in calcium signaling pathway, neuroactive ligand-receptor interaction pathway, and Wnt signaling pathway.

**Conclusion:**

The eight-mRNA signature has greater prognostic value than the ten-lncRNA-based signature for GBM patients based on bioinformatics analysis.

## Background

Glioblastoma (GBM) is the most aggressive primary brain neoplasm [1]. The treatment involves maximal surgical resection along with radiotherapy and chemotherapy [2]. However, the prognosis of GBM patients is dismal that they have a survival of only 12–15 months after the standard treatment, with the 5-year survival rate of 3–5% [1, 3]. Therefore, it is important to develop therapeutic biomarkers for improving survival of GBM patients.

Considerable efforts have been made to identify prognostic gene signatures for GBM. For instance, a three-gene signature of prognostic value for patients with MGMT promoter-methylated GBM is reported by performing bioinformatics analysis [4]. A study finds that a combination of Notch and hypoxia genes is closely associated with survival of GBM patients [5]. Silencing of the signal transducer and activator of transcription-3 (*STAT3*), an important mediator for the subtype of highly aggressive mesenchymal GBM, by a novel aptamer-siRNA chimera (*Gint4.T*) inhibits tumor growth and angiogenesis in a mouse model. Thus, *Gint4.T-STAT3* is suggested as a novel molecule therapy for GBM [6]. In addition, the phosphorylation of STAT3 on Serine727 is identified as a potential prognostic marker for GBM patients [7].

Long non-coding RNAs (lncRNAs) have been focused recently because of its versatile roles in multiple biological processes [8]. Dysregulation of lncRNAs may be potential biomarkers and therapeutic targets for cancer [9]. A study investigates prognostic lncRNAs in GBM by constructing a functional GBM lncRNA-mediated ceRNA network [10]. Besides, using lncRNA expression profiles in GBM patients from The Cancer Genome Atlas (TCGA), a prognostic six-lncRNA signature is identified by applying survival analysis and Cox regression model [11]. Moreover, serum lncRNA *HOTAIR* is proposed as a prognostic biomarker for GBM [12]. Nevertheless, most of these studies have not validated the predictive accuracy of these prognostic signatures and the optimal prognostic model of GBM has not been established.

In the current study, we analyzed the expression of mRNAs and lncRNAs that related to the prognosis in GBM patients from TCGA and built a prognostic prediction model. In addition, accuracy of the predictive model was validated using data in the Chinese Glioma Genome Atlas (CGGA) database. Differential expression on mRNAs were analyzed between high- and low-risk groups based on the optimal prognostic model. Further function and pathway enrichment analyses were performed to provide hints concerning the roles of the prognostic genes in GBM development and physiology.

## Materials and methods

### Data source

The GBM RNA-seq data were downloaded from the TCGA portal (https://gdc-portal.nci. nih.gov/), containing 173 samples (154 primary GBM tissue samples, 14 recurrent samples and 5 normal controls) that sequenced on the platform of Illumina HiSeq 2000 RNA Sequencing (https://tcga.xenahubs.net/download/TCGA.GBM.sampleMap/HiSeqV2.gz). Among them, 151 primary GBM tissue samples that had survival and prognostic information were used as the training set (TCGA set). Meanwhile we downloaded RNA-seq data of 272 gliomas samples named “Part D” (http://cgga.org.cn/download/20191128/CGGA.mRNA_array_301_gene_level.20191128.txt.zip) [13] from the CGGA (http://cgga.org.cn/), 138 of which were histologically confirmed to be GBM, and were then used as the validation set. For the both two sets, the raw data of the Fragments Per Kilobase of exon model per Million mapped fragments (FPKM) in TXT format were downloaded, and then were normalized using the method as demonstrated in a previous study [14], to eliminate the deviation at expression level due to different sequencing platforms or diverse experiment backgrounds.

### Differentially expressed mRNAs and lncRNAs

Briefly, according to RefSeq ID information provided by the training set and the validation set, we annotated mRNAs and lncRNAs of the two sets based on HUGO Gene Nomenclature Committee (HGNC) [15] (http://www.genenames.org/), the database which records information of 4055 lncRNAs and 19,198 protein-coding genes (https://www.genenames.org/cgi-bin/genegroup/download-all). In the training set, samples were classified based on their clinical prognostic information. According to the sample characteristics, the good prognosis samples was defined as patients with overall survival (OS) of more than 6 months and was alive; whereas bad prognosis samples were those with OS less than 6 months and were died. Then, differentially expressed mRNAs (DEGs) and lncRNAs (DE-lncRNAs) were screened between good and bad prognosis patients using the limma package (version 3.34.7) of R. False discovery rate (FDR) < 0.05 and |log_2_ fold change (FC)| > 1 were set as the cutoff for significance. Two-way hierarchical clustering analysis based on centered Pearson correlation algorithm was carried out for these identified DEGs and DE-lncRNAs by pheatmap package [16] (version 1.0.8) of R.

### Prognosis prediction models

In the training set, in order to develop prognosis prediction models, firstly, we determined prognosis-related lncRNAs and mRNAs that were significantly linked to OS from the identified DEGs and DE-lncRNAs by applying univariate Cox regression model and log-rank test. A gene or a lncRNA with log-rank *p* < 0.05 was considered as significant. These identified prognosis-related DEGs and DE-lncRNAs were then used to fit L1-penalized (LASSO) Cox-PH regression model [17] for selection of the optimal predictive mRNAs and lncRNAs with penalized package (version 0.9–50) of R (http://bioconductor.org/packages/penalized/). The optimal parameter of ‘lambda’ in the selection model was calculated via the cross-validation likelihood (cvl) method for 1000 times.

### Prognosis prediction models based on expression status of prognostic RNAs

X-Tile tool [18] was applied to calculate the optimal cutoff point for expression levels of the aforementioned prognostic mRNAs and lncRNAs based on patients’ survival. Monte-Carlo *P* < 0.05 were set as the threshold for the cutoff points. Then the RNA expression status of a sample was defined based on the RNA’s cutoff value: if expression level of a RNA is above the cutoff value (high expression), its expression status is equal to 1; otherwise, the expression level below the cutoff value (low expression), the RNA expression status of is equal to 0. Combing the expression status with the Cox regression coefficients of these prognostic RNAs, the sample risk assessment system was constructed, and the risk score of each sample was obtained using the following formula:

$$ \mathrm{Status}\ \mathrm{Risk}\ \mathrm{Score}=\sum {\upbeta}_{\mathrm{RNAn}}\times {\mathrm{Status}}_{\mathrm{RNAn}}. $$

β_RNAn_ indicates Cox-PH coefficient of a RNA; Status _RNAn_ indicates expression status of a RNA.

### Prognosis prediction model based on expression levels of prognostic RNAs

Using expression levels of the DEGs and DE-lncRNAs, and their Cox regression coefficients, the prognostic scoring models were established with the following formula:

$$ \mathrm{Expression}\ \mathrm{Risk}\ \mathrm{Score}=\sum {\upbeta}_{\mathrm{RNAn}}\times {\mathrm{Exp}}_{\mathrm{RNAn}}. $$

β _RNAn_ and Exp _RNAn_ represents Cox-PH coefficient and expression level of a RNA, respectively. For lncRNA, the expression unit is FPKM.

To detect the predictive ability of different prognostic scoring models, they were tested on the training set and the validation set, separately. According to median risk score, each set was classified into a high-risk group and a low-risk group. Thereafter, Kaplan-Meier (KM) method was used to assess the survival curves between groups. Additionally, the accuracy for survival prediction using these models were evaluated by area of the receiver operating characteristic curve (AUC). By integrating the parameters of the four models in both training set and validation set, the optimal prognosis prediction model was selected.

### Nomogram survival rate model for independent prognostic factor

Using the univariate and multivariate regression analysis in the survival package of R (version 2.41–1), the prognostic factors in the training set were selected with the significance threshold of log-rank *p* < 0.05.

To further investigate the correlations between the independent prognostic factors and the survival, we combined the identified independent prognostic factors with the predicted risk information in the prediction prognosis model to construct a nomogram 1-year or 3-year survival rate model [19] by using rms package of R. The actual and predicted probabilities of 1-year OS and 3-year OS were compared using calibration plots. *P* < 0.05 suggests statistical significance.

### Function analysis

All samples in the training set were divided into high-risk and low-risk groups according to the risk score obtained from the optimal prognosis prediction model. Then, DEGs between the two risk groups were analyzed using the limma package in R (version 3.34.7), with the cutoff of FDR < 0.05 and |log_2_FC| > 0.263. The FC here was set as 1.2 because if it was set as 2 as aforementioned, the DEG numbers were small, which would impede the following enrichment analysis.

Afterwards, using the clusterProfiler package [20] (version 3.6.0) of R, we performed gene ontology (GO) function and Kyoto Encyclopedia of Genes and Genomes (KEGG) pathway enrichment analyses to obtain the biological processes and KEGG signaling pathways that were significantly enriched with these identified DEGs. Statistical significance was set at *p* < 0.05.

## Results

### Identification of DEMs and DELs

**Fig. 1:**
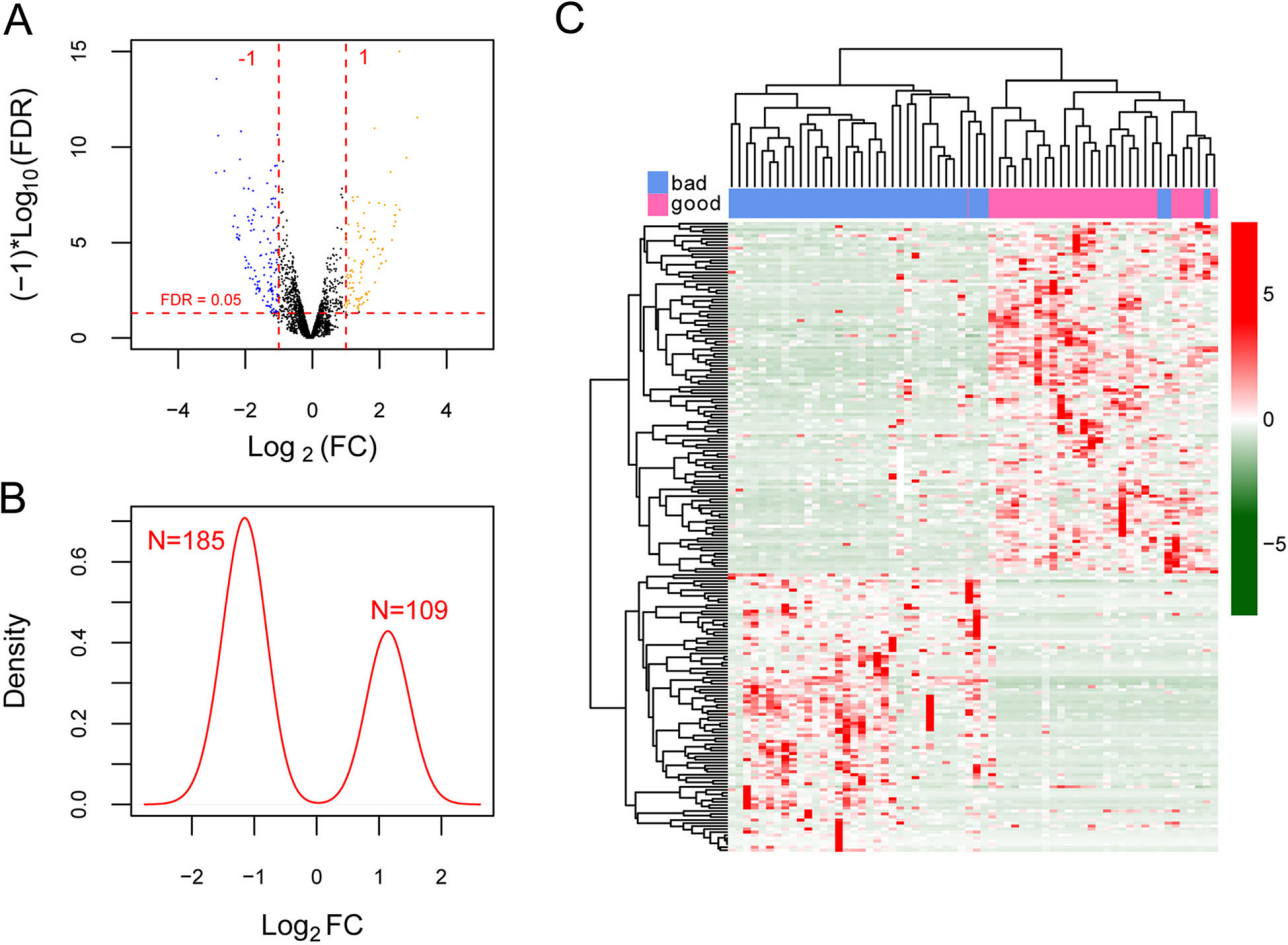
Graphic representation of differentially expressed RNAs (DEGs) between good prognosis and bad prognosis patients. **a**, volcano plot of effect size (log_2_FC) and -log^10^(FDR) of DEGs. Blue and orange round spots stand for down- and up-regulated RNAs, respectively. Two red vertical dash lines signal |logFC| > 1, and red horizontal dash line signals FDR < 0.05. **b**, Kernel density plot for DEGs. **c**, a heatmap illustrating two-way hierarchical clustering of DEGs. Horizontal blue and pink bars indicate good prognosis and bad prognosis samples, respectively. Bad: bad prognosis patients; good: good prognosis patients

Following data annotation, we obtained 17,299 mRNAs and 770 lncRNAs overlapped by the TCGA set and the validation set. Based on predefined classification criteria, there identified 27 good prognosis samples and 37 bad prognosis samples in the TCGA set. In total, 261 mRNAs and 33 lncRNAs were differentially expressed between good prognosis and bad prognosis samples of the training set, including 182 down-regulated and 79 up-regulated mRNAs, 3 down-regulated and 30 up-regulated lncRNAs. To show whether FDR value and |log_2_FC| conformed to logic with different test, we illustrated these results in a volcano plot (Fig. 1a) and a kernel density plot (Fig. 1b). Two-way hierarchical clustering of these DEGs and DE-lncRNAs was depicted in a heatmap (Fig. 1c). The results showed that good prognosis samples could be well distinguished from bad prognosis samples based on expression patterns of these DEGs and DE-lncRNAs. The specific mRNA and lncRNA names are provided in the appendix 1.

### The prognostic model based on expression levels of 8 mRNAs was superior to the ten-lncRNA-based prognostic model

Combining the DEGs and DE-lncRNAs with the prognostic information, the univariate Cox regression analysis identified 21 of the 261 DEGs and 16 of the 33 DE-lncRNAs were significantly correlated with OS. These prognosis-related DEGs and DE-lncRNAs were then used to fit the LASSO Cox-PH regression model, respectively, to select the optimal RNA set relating to prognosis. As defined in the method section, the lambda value was determined by conducting 1000 cvl. A panel of 8 optimal DEG-set was obtained at the maximum value of cvl (− 557.1395) when lambda was 7.1358; and 10 optimal DE-lncRNA set were obtained (lambda = 3.2821, cvlmax = − 558.6265) (Table 1). The eight mRNAs were acid sensing ion channel subunit family member (*ASIC*), chorionic gonadotropin subunit beta (*CGB*), claudin (*CLDN*)*16*, keratin (*KRT*)*13*, hyaluronan and proteoglycan link protein (*HAPLN*)*4*, lipocalin (*LCN*)9, sclerostin (*SOST*), and tensin (*TNS*)*4*. The ten lncRNAs consisted of homeobox (HOXB)–antisense (AS)3 (*HOXB-AS3*), *LGALS8-AS1*, Long intergenic non-protein coding (LINC)00032 (*LINC00032*), *LINC00311*, *LINC00494*, *LINC00544*, *LINC00589*, *LINC00626*, *MEIS1-AS3* and *VAV3-AS1*.

**Table 1:** Ten lncRNAs and eight mRNAs selected as prognostic RNAs

Type	ID	coefficient	*P*-value	HR	95%CI	X-tile cutoff
lncRNA	HOXB-AS3	0.074	0.007	1.350	1.086–1.676	0.87
	LGALS8-AS1	0.122	0.014	1.292	1.053–1.584	0.20
	LINC00032	−0.003	0.020	0.795	0.656–0.963	0.20
	LINC00311	0.060	0.001	1.481	1.173–1.868	−2.01
	LINC00494	−0.004	0.013	0.842	0.672–0.952	−0.14
	LINC00544	0.021	0.021	1.265	1.035–1.543	0.02
	LINC00589	0.067	0.019	1.147	0.934–1.407	0.00
	LINC00626	−0.005	0.018	0.770	0.619–0.956	0.00
	MEIS1-AS3	−0.053	0.040	0.803	0.650–0.989	0.15
	VAV3-AS1	0.104	0.042	1.225	0.998–1.502	−0.09
mRNA	ASIC5	0.065	0.035	1.246	1.016–1.528	−1.67
	CGB7	0.072	0.003	1.405	1.124–1.756	−0.47
	CLDN16	0.123	0.006	1.372	1.096–1.717	−2.39
	HAPLN4	0.143	0.000	1.518	1.228–1.877	−2.03
	KRT13	0.084	0.002	1.362	1.123–1.652	−0.01
	LCN9	0.059	0.012	1.300	1.060–1.595	−0.22
	SOST	0.033	0.018	1.148	1.038–1.404	0.60
	TNS4	0.028	0.041	1.231	1.009–1.503	−0.14

*HR* Hazard ratio, *CI* Confidential interval

As aforementioned,, X-Tile bio-informatics tool was used to explore the correlations between the above optimal sets with the prognosis, and to get the cutoff value of each RNA. After obtaining the expression status in each sample, a prognosis risk prediction model based on expression status and Cox-PH coefficients of the ten prognostic lncRNAs was established as follows (model 1):

lncRNA status Risk Score = (0.074) * Status_HOXB-AS3_ + (0.122) * Status_LGALS8-AS1_ + (− 0.003) * Status_LINC00032_ + (0.060) * Status_LINC00311_ + (− 0.004) * Status_LINC00494_ + (0.021) * Status_LINC00544_ + (0.067) * Status_LINC00589_ + (− 0.005) * Status_LINC00626_ + (− 0.053) * Status_MEIS1-AS3_ + (0.104) * Status _VAV3- AS1_.

The prognostic prediction model based on the eight prognostic mRNAs was developed as follows (model 2):

mRNA status Risk Score = (0.065) * Status_ASIC5_ + (0.072) * Status_CGB7_ + (0.123) * Status_CLDN16_ + (0.143) * Status_HAPLN4_ + (0.084) * Status_KRT13_ + (0.123) * Status_LCN9_+ (0.143) * Status_SOST_ +(0.084) * Status _TNS4_.

The model based on Cox-PH coefficients and expression levels of the eight prognostic mRNAs were built as follows (model 3):

lncRNA Expression Risk Score = (0.074) * Exp_HOXB-AS3_ + (0.122) * Exp_LGALS8-AS1_ + (− 0.003) * Exp_LINC00032_ + (0.060) * Exp_LINC00311_ + (− 0.004) * Exp_LINC00494_ + (0.021) * Exp_LINC00544_ + (0.067) * Exp_LINC00589_ + (− 0.005) * Exp_LINC00626_ + (− 0.053) * Exp_MEIS1-AS3_ + (0.104) * Exp_VAV3-AS1_.

Similarly, the model based on the ten lncRNAs was established as follows (model 4):

mRNA Expression Risk Score = (0.065) *Exp_ASIC5_ + (0.072) * Exp_CGB7_ + (0.123) * Exp_CLDN16_ + (0.143) * Exp_HAPLN4_ + (0.084) * Exp_KRT13_+ (0.123) * Exp_LCN9_ + (0.143) * Exp_SOST_ + (0.084) * Exp _TNS4_.

**Fig. 2:**
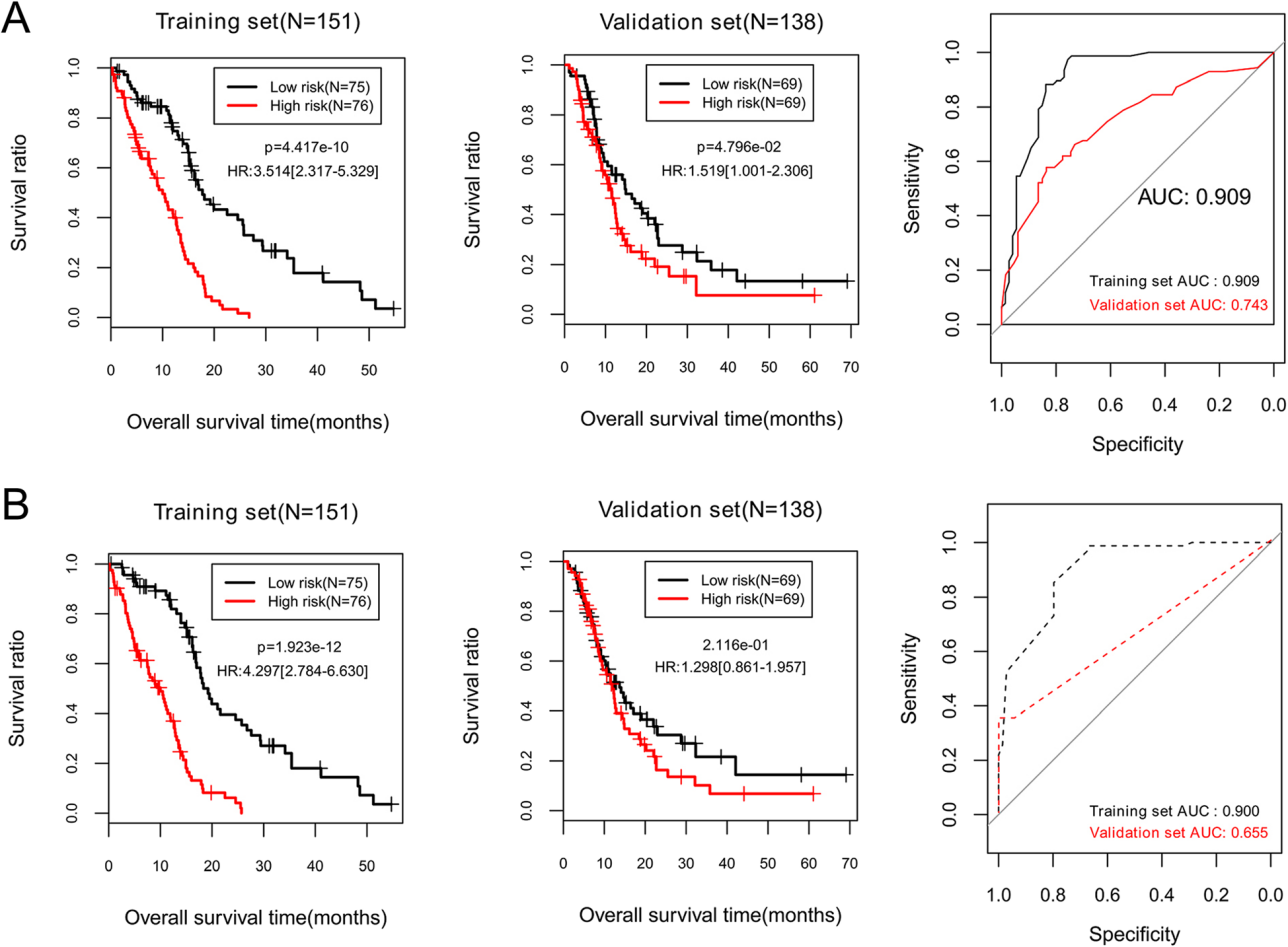
Kaplan-Meier survival curves and the receiver operating characteristic curve (AUC) to evaluate predictive performance of the prognosis prediction models based on expression status of the selected biomarkers in the training set and the validation set. **a**, selected ten lncRNAs; **b**, selected eight mRNAs. All patients in each set are classified by each prognostic model into a high-risk group and a low-risk group

**Fig. 3:**
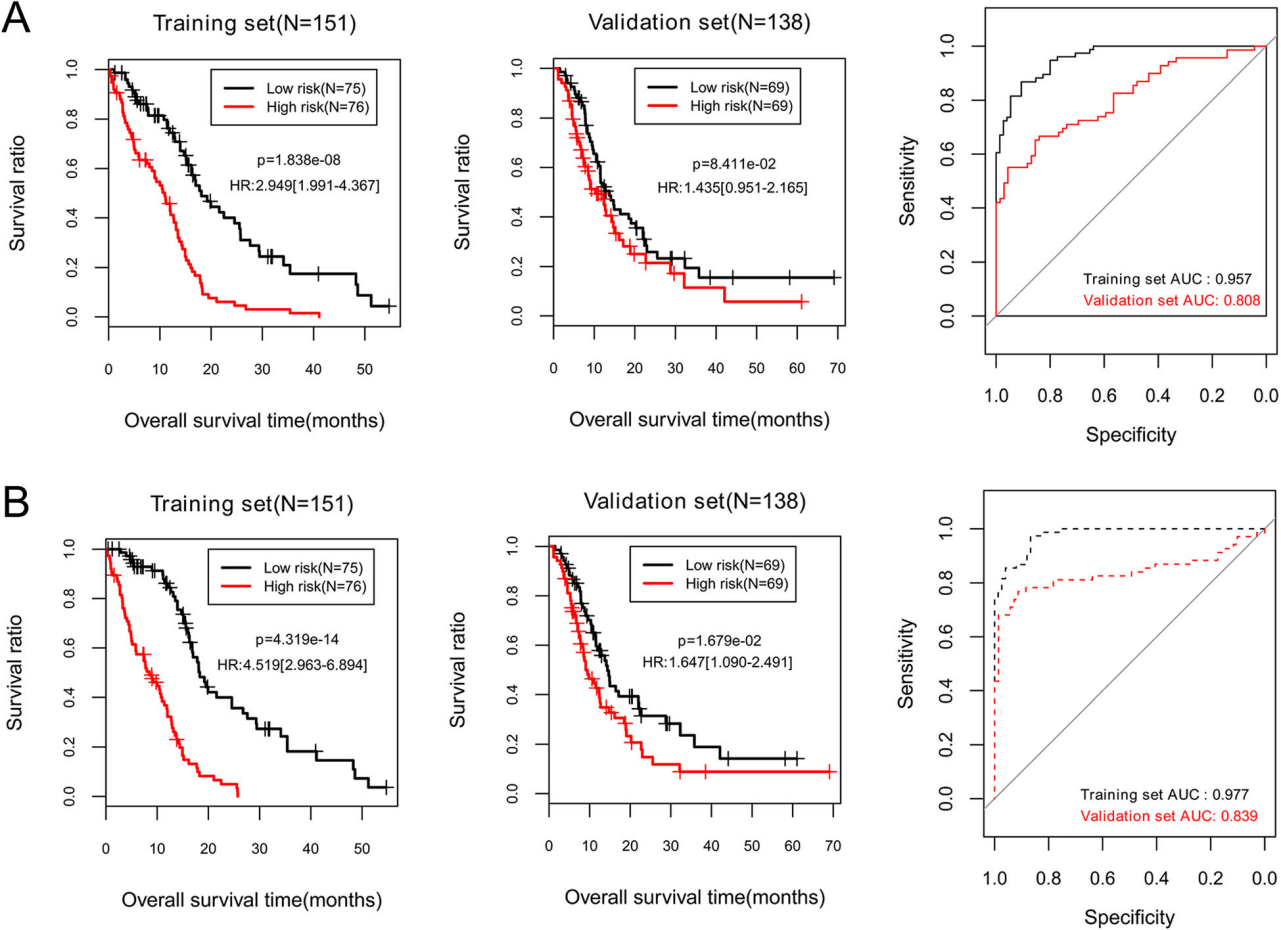
Kaplan-Meier survival curves and the receiver operating characteristic curve (AUC) to assess predictive ability of the prognosis prediction models based on expression levels of the selected biomarkers in the training set and the validation set. **a**, selected ten lncRNAs; **b**, selected eight mRNAs. Each set is separated by each prognostic model into a high-risk group and a low-risk group

To compare prognostic performances of the four prognosis prediction models, all patients in the training set or the validation set were separated into a high-risk group and a low-risk group by risk score obtained from each model, separately (Figs. 2-3). OS of the two risk groups were analyzed using KM plot and log-rank test, and predictive ability of each model was measured by AUC curves. Log-rank p and AUC values were summarized in Table 2.

**Table 2:** Predictive performances of four prognosis prediction models based on expression levels or status of lncRNAs or mRNAs

Type	Status model				Expression model			
	LogRank p-value		AUC value		LogRank p-value		AUC value	
	Training set	Validation set	Training set	Validation set	Training set	Validation set	Training set	Validation set
lncRNA	4.42 × 10^−10^	0.0480	0.907	0.743	1.84 × 10^− 08^	0.0841	0.957	0.808
mRNA	1.92 × 10^−12^	0.2116	0.900	0.655	4.32 × 10^−14^	0.0168	0.977	0.839

*AUC* Area of the receiver operating characteristic curve

Among the four prognostic models, the model based on expression levels of the eight mRNAs generated the lowest *p*-values (training set, *p* = 4.32 × 10^− 14^; validation set, *p* = 0.01679) and the highest AUC values (training set, AUC = 0.977; validation set, AUC = 0.839) for the training set and the validation set (Table 2, Fig. 3b). These results indicate a better predictive capability of this prognostic model (model 3) than others, which was then selected for further analyses.

### Building nomogram based on prognostic clinical factors and the eight-mRNA signature

**Fig. 4:**
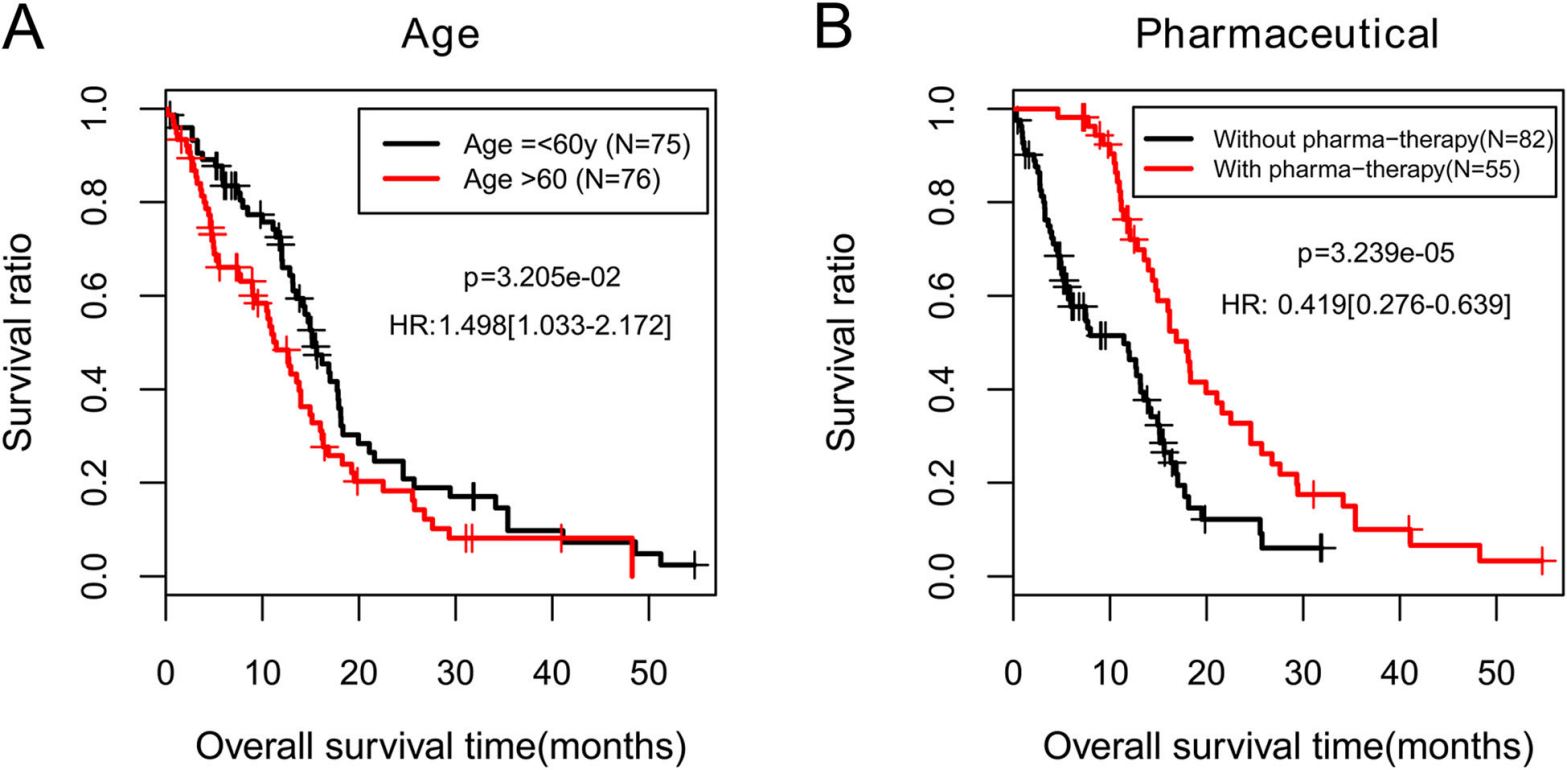
Kaplan-Meier curves for overall survival of patients in the training set classified by clinical features. **a**, age; **b**, pharmaceutical therapy. All patients in the training set are divided by age or pharmaceutical therapy into two subgroups, respectively. The patients younger than or equal to 60 years have significantly longer overall survival time than the patients older than 60 years (*p* = 3.205 × 10^−2^). Markedly better survival is observed in the patients with pharmaceutical therapy compared to the patients without pharmaceutical therapy (*p* = 3.239 × 10^−5^)

**Fig. 5:**
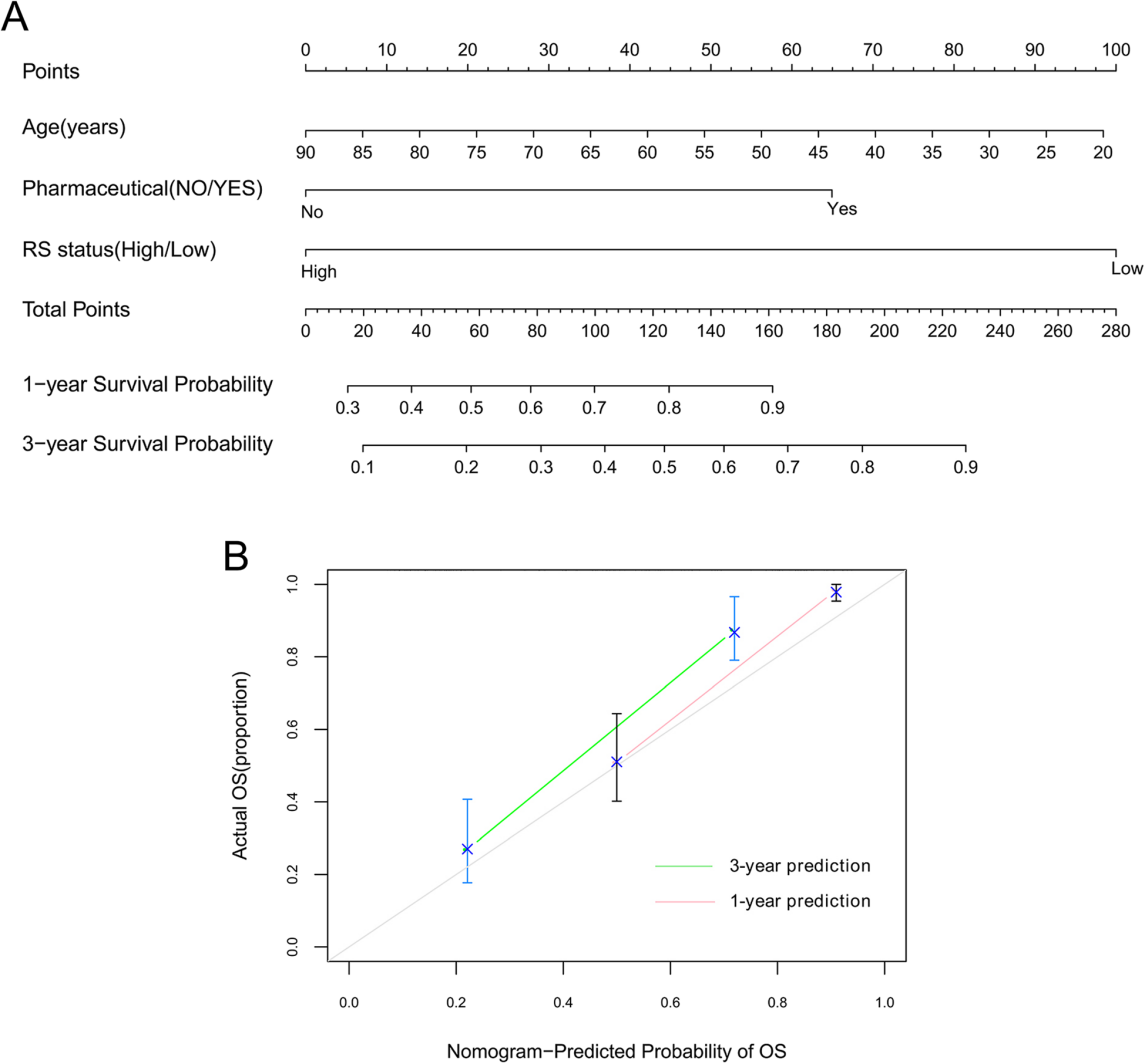
A nomogram incorporating risk score based on expression levels of eight mRNAs, age and pharmaceutical therapy for predicting survival of GBM patients. **a**, the sum of points for each variable value is located on Total Point axis, and used to determine likelihood of 1-year and 3-year overall survival of each individual patient. **b**, calibration plots of nomogram for predicting 1-year and 3-year overall survival

As shown in Table 3 and Fig. 4, we found that age and pharmaceutical therapy were independent prognostic factors in the training set. Combining expression risk score based on the eight-mRNA signature with age and pharmaceutical therapy, we constructed a nomogram to improve predictive accuracy (Fig. 5a). As shown in calibration plots (Fig. 5b), consist results were observed between predicted and actual 1- and 3-year OS, respectively.

**Table 3:** Determination of prognostic clinical factors

Clinical characteristics	Training set (*N* = 151)	Uni-variables cox			Multi-variables cox		
		HR	95%CI	P-value	HR	95%CI	*P*-value
Age (years, mean ± SD)	59.82 ± 13.67	1.029	1.014–1.045	1.966 × 10^− 04^	1.022	1.005–1.039	1.310 × 10^−02^
Gender (Male/Female)	97/54	0.899	0.613–1.320	5.886 × 10^−01^	–	–	–
Chemo-therapy (Yes/No/−)	45/89/17	0.603	0.397–0.915	1.625 × 10^− 02^	1.791	0.895–3.585	9.960 × 10^− 02^
Drug-therapy (Yes/No/−)	20/113/18	0.729	0.431–1.234	2.377 × 10^−01^	–	–	–
Immuno-therapy (Yes/No/−)	2/131/18	0.555	0.136–2.264	4.050 × 10^−01^	–	–	–
Pharmaceutical-therapy (Yes/No/−)	55/82/14	0.419	0.276–0.639	3.239 × 10^−05^	0.335	0.169–0.663	1.700 × 10^−03^
Targeted molecular-therapy (Yes/No/−)	18/115/18	0.889	0.519–1.523	6.701 × 10^−01^	–	–	–
Radiotherapy (Yes/No/−)	20/117/14	0.611	0.346–1.078	8.575 × 10^−02^	–	–	–
Risk status (High/Low)	75/76	4.519	2.963–6.894	4.319 × 10^−14^	4.179	2.631–6.640	1.390 × 10^−09^
Dead (Death/Alive/−)	115/36	–	–	–	–	–	–
Overall survival time (months, mean ± SD)	13.26 ± 10.82	–	–	–	–	–	–

*SD* Standard deviation, *HR* Hazard ratio, *CI* Confidential interval

### Function analysis of the prognostic eight-gene signature

**Fig. 6:**
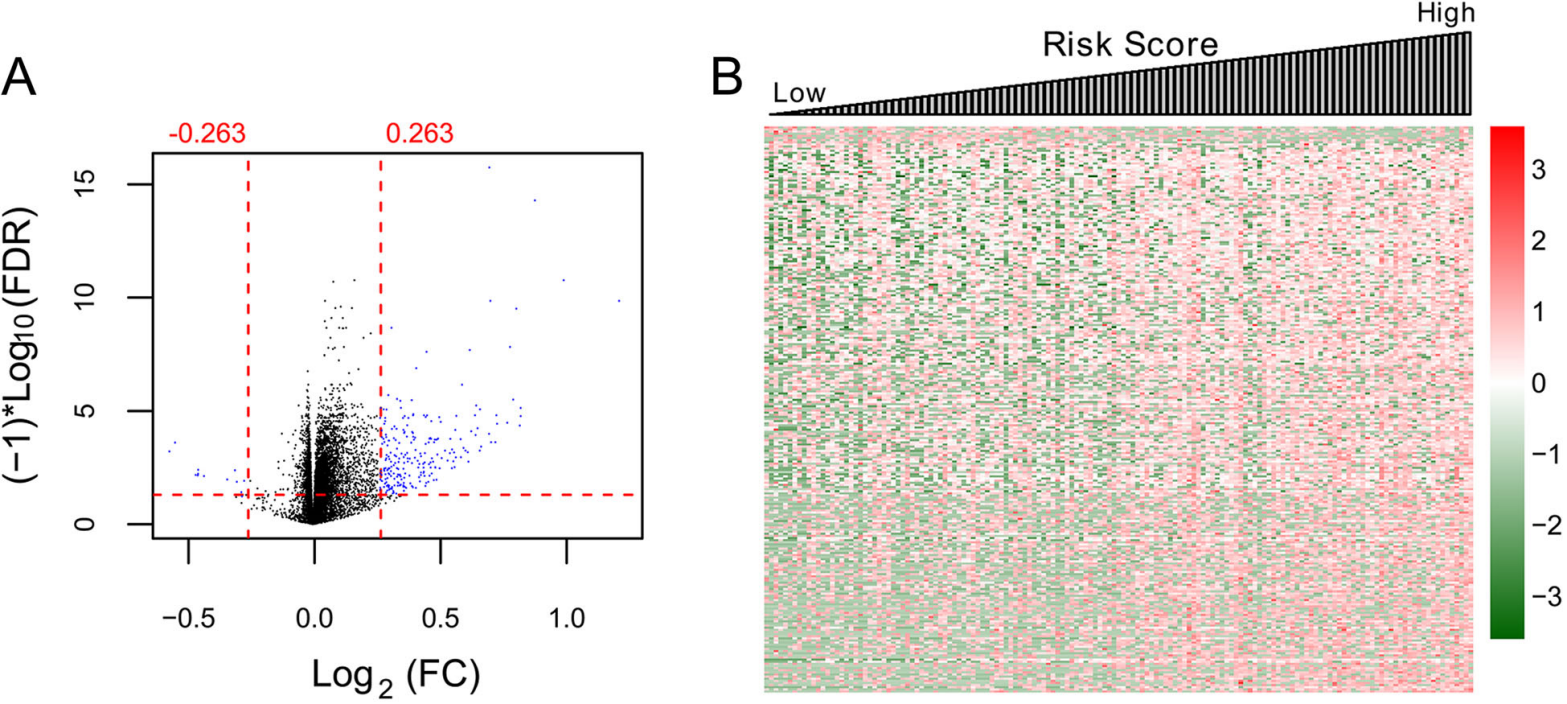
Graphic demonstrations of DEGs between high and low risk subgroups of training set by the eight-mRNA signature. **a**, volcano plot of effect size (log_2_FC) and -log^10^(FDR) of the 266 DEGs. Blue and black spots represent significant DEGs and non-DEGs, respectively. **b**, risk score and expression levels of DEGs for patients in the high- and low-risk subgroups

We used the expression risk score of the eight-mRNAs in the mRNA expression based model (model 3) to classify samples in the TCGA set into high-risk group and low-risk group. We then screened DEGs between the two risk groups. As a result, 11 down-regulated and 255 up-regulated genes (selection criteria: FDR < 0.05 and |log2FC| > 0.263) were found between groups (Fig. 6). Detailed genes are provided in appendix 2. These genes were significantly related to 25 GO biological processes, such as cellular metal ion homeostasis and cellular calcium ion homeostasis (Table 4). Moreover, 5 KEGG signaling pathways functionally involved these DEGs, consisting of neuroactive ligand-receptor interaction, tight junction, Wnt signaling pathway, calcium signaling pathway, and cytokine-cytokine receptor interaction (Table 4).

**Table 4:** Significant GO biological processes and KEGG pathways

Category	Term	Count of genes	*P*-value
Biology Process	Cellular metal ion homeostasis	12	6.570 × 10^− 05^
	Metal ion homeostasis	12	9.840 × 10^−05^
	Cellular cation homeostasis	13	1.590 × 10^−04^
	Cellular calcium ion homeostasis	11	1.760 × 10^− 04^
	Calcium ion homeostasis	11	2.200 × 10^−04^
	Cellular di-, tri-valent inorganic cation homeostasis	12	2.410 × 10^−04^
	Di-, tri-valent inorganic cation homeostasis	12	3.740 × 10^−04^
	Cell-cell signaling	20	4.350 × 10^−04^
	Cation homeostasis	13	4.710 × 10^−04^
	Regulation of system process	13	9.290 × 10^−04^
	Ectoderm development	10	1.434 × 10^−03^
	Cellular ion homeostasis	13	4.521 × 10^− 03^
	Cellular chemical homeostasis	13	5.122 × 10^−03^
	Chemical homeostasis	15	8.944 × 10^−03^
	Ion homeostasis	13	8.989 × 10^−03^
	Response to wounding	15	1.186 × 10^−02^
	Cellular homeostasis	13	2.288 × 10^−02^
	Behavior	13	2.390 × 10^− 02^
	Multicellular organism reproduction	13	3.075 × 10^−02^
	Reproductive process in a multicellular organism	13	3.075 × 10^−02^
	Defense response	15	3.669 × 10^−02^
	Gamete generation	11	3.984 × 10^−02^
	Homeostatic process	17	4.436 × 10^−02^
	G-protein coupled receptor protein signaling pathway	23	4.513 × 10^−02^
	Transmission of nerve impulse	10	4.589 × 10^−02^
KEGG Pathway	Neuroactive ligand-receptor interaction	9	7.151 × 10^−04^
	Tight junction	4	1.892 × 10^−02^
	Wnt signaling pathway	4	2.391 × 10^−02^
	Calcium signaling pathway	4	3.157 × 10^−02^
	Cytokine-cytokine receptor interaction	5	3.380 × 10^−02^

Count of genes, the total number of genes significantly involved in a GO biological process or pathway; GO, gene ontology; KEGG, Kyoto Encyclopedia of Genes and Genomes pathway enrichment

## Discussion

Prognosis of GBM patients varies greatly due to heterogeneity of the disease, demanding development of prognostic molecular indicators to improve outcome of GBM patients [21]. In the present study, we identified a ten-lncRNA signature and an eight-mRNA prognostic signature (*ASIC5*, *CGB7*, *CLDN16*, *HAPLN4*, *KRT13*, *LCN9*, *SOST* and *TNS4*) of GBM. Then, based on these signature sets, four prognosis prediction models were built using their expression levels or expression status, and the prognostic model based on expression levels of the eight- mRNA was superior to the others in predicting survival of GBM patients. To our knowledge, this is the first study that compares mRNA and lncRNA signatures for predicting outcome of GBM patients. The eight-mRNA signature was capable to classify GBM patients into subgroups with significantly different OS, showing greater prognostic power. Results of validation set revealed good reproducibility and robustness of the eight-mRNA signature. Application of this eight-mRNA signature would help clinicians to select patients at high risk of death, thereby facilitating development of individualized therapies for GBM patients.

Of the eight mRNAs, *ASIC5* is a member of ASIC channels that primarily locate in the central and peripheral nervous system. ASIC channels participate in diverse processes during tumor development, such as apoptosis, proliferation and migration, and may be potential therapeutic targets for cancer therapies [22]. Importantly, ASICs are correlated with malignant gliomas. *ASIC1a* and *ASIC3* are expressed in GBM stem cell lines [23], and knockdown of *ASIC1* in vitro inhibits the cell mobility and migration of GBM [24]. However, function of *ASIC5* has not been revealed. Based on our study, expression of *ASIC5* might be associated with prognosis of GBM.

*CGB7* gene encodes the beta 7 subunit of chorionic gonadotropin. It has been demonstrated that human chorionic gonadotropin β promotes development of GBM [25]. However, there are few studies reporting the correlation of this gene with prognosis. Our study might provide novel insights into GBM prognosis with this gene.

Claudin-16 encoded by *CLDN16* is a component of tight junction strands. Abnormal tight junction functions have been established as a hallmark of human carcinomas [26]. Reduced expressions of *CLDN1* and *CLDN5*, two family members of *CLDN16*, are associated with the progression of GBM multiforme [27]. According to our results, altered expression of *CLDN16* might be also related to the development of GBM, and the dysregulation might indicate prognosis of the disease.

*HAPLNs* are involved in forming and controlling perineuronal matrix in the adult brain, thus regulating neuronal function and plasticity [28]. *HAPLN4* is obviously decreased in the parenchyma of malignant gliomas, and *HAPLN4* expression promotes glioma cell adhesion and migration [29]. *KRT13* encodes keratin 13 that is a type I cytokeratin (CK), and it is also named as *CX13*. Reportedly, 4% of GBM patients are estimated to be immunoreactive for cytokeratin, the reaction, however, is normally weak [30]. Although there is evidence that expressions of other CK members including *CK34BE12*, *CK5*, *CK6*, *CK7*, *CK8*, *CK14*, *CK18*, *CK19* and *CK20* are absent in GBM [31], there are rare reports on the roles of *CK13* in GBM. Sclerostin encoded by *SOST* is produced primarily by osteocyte, and may be implicated in promoting tumor growth, bone metastasis and osteolysis in breast cancer [32]. Sclerostin is emerging as a potential target to treat cancer-related bone diseases [33]. Tensins localized in the cytoplasmic tails of integrins at focal adhesions are critical for cell adhesion, migration and invasion [34]. *TNS4* plays an important part in stability of receptor tyrosine kinase, thus regulating survival and proliferation of carcinoma cells [35]. High expression of *TNS4* is associated with a poor prognosis in many cancer types, such as gastric cancer and lung adenocarcinoma [36, 37]. However, studies on this gene in GBM have not been reported. Lipocalins are small extracellular proteins that participate in cell regulation, differentiation and proliferation [38]. Downregulation of *LCN2* involves in the chemoresistance in GBM cells [39]. However, studies on the role of *LCN9* in cancers are rare.. Our results might provide a hint for the future studies on the above genes. To gain insights into the functional roles of the eight prognostic mRNAs in the molecular mechanisms of GBM, GO function and KEGG pathway enrichment analyses were carried out for the DEGs between the predicted high-risk and low-risk groups of the training set by the eight-mRNA signature. The results suggested that these genes were functionally associated with cellular metal ion homeostasis, cellular calcium ion homeostasis, calcium signaling pathway, neuroactive ligand-receptor interaction pathway, and Wnt signaling pathway. Increasing evidence has indicated that neuroactive ligand-receptor interaction pathway may play an important role in the biology of GBM [40, 41]. Oncogenic roles of Wnt signaling pathway in GBM has been demonstrated previously, and the pathway is also involved in GBM stem cell maintenance and invasion [42]. Calcium signaling is closely related to tumorigenesis and progression of GBM [43]. These findings collectively suggest the eight genes might be important regulators in GBM progression and might function via the involvement of the above processes and pathways. Further functional characterization of the eight-mRNA signature would be beneficial to unraveling the underlying mechanism of GBM.

LncRNAs are research hotspots currently, and several lncRNAs are identified as potential prognostic factors in GBM or glioma, such as *TP73-AS1* [44], *LINC00599* [45] and *HOXA11-AS* [46]. However, due to heterogeneity of the disease, it is insufficient to use a single lncRNA biomarker. A panel of ten-lncRNA signature relating to prognosis was identified in our study. Among them, *LGALS8* is reported to promote cell proliferation in a GBM model [47]; *VAV3* is highly expressed in breast cancer and GBM, and the overexpression indicates a poor prognosis of breast cancer [48]; *LINC00311* is overexpressed in ankylosing spondylitis and promotes the proliferation and differentiation of osteoclasts [49, 50]. The remaining lncRNAs are rarely reported in previous studies. Our findings using them as a whole panel might provide novel insights into prognostic prediction of GBM. Interestingly, based on our study, the performance of the predictive prognosis with the ten-lncRNA signature was inferior to that using the eight-mRNA signature.

Although we have identified novel signatures for the prediction of GBM prognosis, and validated the accuracy using these predictive models in the validation set, several limitations remained in the study. First, the sample size was small in both of the training set and validation set, making a relatively weak statistic power. In addition, the prognostic value of these signatures needs to be validated in the GBM patients. Nevertheless, our study has predictive values and provides a foundation for future studies.

## Conclusion

We develop an eight-mRNA and a ten-lncRNA prognostic signatures that are able to classify GBM patients into subgroups with different prognostic risks. The eight-mRNA signature is superior to the ten-lncRNAs signature for prognostic risk classification of GBM patients. These findings would aid in improving outcome of GBM patients. Further validation of this eight-mRNA signature on large cohorts of GBM patients is warranted prior to clinical application.

## Supplementary information

**Additional file 1.**

**Additional file 2.**

## Data Availability

Raw data in this study were from the public databases, RNA sequencing data of 173 samples (154 primary GBM tissue samples, 14 recurrent samples and 5 normal controls) that sequenced on the platform of Illumina HiSeq 2000 RNA Sequencing (https://tcga.xenahubs.net/download/TCGA.GBM.sampleMap/HiSeqV2.gz) were from TCGA (https://gdc-portal.nci. nih.gov/) database; the RNA-seq data of 272 gliomas samples named “Part D” (http://cgga.org.cn/download/20191128/CGGA.mRNA_array_301_gene_level.20191128.txt.zip) were extracted from CGGA (http://cgga.org.cn/) database, and protein annotation information was from HGNC (http://www.genenames.org/) database;, and the annotation files were also downloaded (https://www.genenames.org/cgi-bin/genegroup/download-all).

## Abbreviations

GBM: Glioblastoma
DEGs: Differentially expressed mRNAs
DE-lncRNAs: lncRNAs
PH: Proportional-hazards
lncRNAs: Long non-coding RNAs
TCGA: The Cancer Genome Atlas
ROC: Receiver operating characteristic
GO: Gene ontology
CGB: Chorionic gonadotropin subunit beta
